# Enhanced Natamycin production by *Streptomyces natalensis* in shake-flasks and stirred tank bioreactor under batch and fed-batch conditions

**DOI:** 10.1186/s12896-019-0546-2

**Published:** 2019-07-16

**Authors:** Elsayed Ahmed Elsayed, Mohamed A. Farid, Hesham A. El-Enshasy

**Affiliations:** 1 Bioproducts Research Chair, Zoology Department, Faculty of Science, King Saud University, 11451 Riyadh, Kingdom of Saudi Arabia; 2 Chemistry of Natural and Microbial Products Department, National Research Centre, Dokki, Cairo, Egypt; 3 Institute of Bioproduct Development (IBD), Universiti Teknologi Malaysia (UTM), 81130 UTM Skudai, Malaysia; 4 City of Scientific Research and Technology Application, New Burg Al Arab, Alexandria, Egypt

**Keywords:** Natamycin, Catabolite repression, Degradation, *Streptomyces natalensis*, Feeding, Bioreactor

## Abstract

**Background:**

Natamycin is an antifungal polyene macrolide antibiotic with wide applications in health and food industries. Currently, it is the only antifungal food additive with the GRAS status (Generally Regarded as Safe).

**Results:**

Natamycin production was investigated under the effect of different initial glucose concentrations. Maximal antibiotic production (1.58 ± 0.032 g/L) was achieved at 20 g/L glucose. Under glucose limitation, natamycin production was retarded and the produced antibiotic was degraded. Higher glucose concentrations resulted in carbon catabolite repression. Secondly, intermittent feeding of glucose improved natamycin production due to overcoming glucose catabolite regulation, and moreover it was superior to glucose-beef mixture feeding, which overcomes catabolite regulation, but increased cell growth on the expense of natamycin production. Finally, the process was optimized in 7.5 L stirred tank bioreactor under batch and fed-batch conditions. Continuous glucose feeding for 30 h increased volumetric natamycin production by about 1.6- and 1.72-folds in than the batch cultivation in bioreactor and shake-flasks, respectively.

**Conclusions:**

Glucose is a crucial substrate that significantly affects the production of natamycin, and its slow feeding is recommended to alleviate the effects of carbon catabolite regulation as well as to prevent product degradation under carbon source limitation. Cultivation in bioreactor under glucose feeding increased maximal volumetric enzyme production by about 72% from the initial starting conditions.

## Background

Natamycin is a polyene antibiotic produced by different *Streptomyces* species in aerobic submerged cultivations. It is also known as pimaricin, tennecetin or natacyn [1, 2]. It belongs to the tetraene macrolide antibiotics due to the presence of 4 conjugated double bonds in its lactone ring, which is in turn attached to a mycosamine unit [3, 4]. Major producing strains are *S. natalensis, S. gilvosporeus, S. chattanogenesis* and *S. lydicus* [5, 6]. Natamycin exhibits its antimicrobial effect by disrupting the ergosterol content in fungal cell walls, and therefore is characterized by its wide spectrum of antifungal activity against yeast, molds and protozoa [7, 8]. Accordingly, natamycin has found a wide range of applications in the treatment of skin infections, ophthalmic mycoses, oral candidiasis, vaginal candidiasis, bronchopulmonary infections and many systemic fungal infections [9, 10]. Moreover, natamycin is widely applied in many food industries as a preservative due to its low toxicity to mammalian cells compared to other antimicrobial compounds, and the unlikelihood development of bacterial resistance [1, 11]. It is considered one of the most important antibiotics added as food preservative approved by the US-FDA and the European Union. Moreover, it is currently the only antifungal food additive with a GRAS (Generally Regarded as Safe) status [12]. It is used in many cheese and sausage ripening processes, food packages or coatings due to its inability to diffuse into the product. It is also used to prevent fungal growth in beverages as well as in crop protection [13, 14].

The production of natamycin is usually carried out in submerged cultivations. Different strategies have been used to maximize natamycin production. These include optimization of medium composition and cultivation parameters [2, 5, 15, 16], engineering of the biosynthetic pathway [17], random mutation and selection [18] and genome shuffling and chromosomal integration [19]. Furthermore, addition of short-chain carboxylic or fatty acids has been found to improve natamycin production [5, 20]. The production process of antibiotics mainly depends on applying batch or fed-batch modes [21–24]. Fed-batch is characterized by feeding one or more of the components of the production medium, usually the limiting components, to avoid problems associated with their depletion from medium. Pathway engineering and genetic manipulation greatly improved natamycin production, where the improved strains produced about about 3.3 and 8.2 g/L natamycin in shake-flask and bioreactor levels, respectively [17, 19]. On the other hand, optimization of medium composition and cultivation parameters in shake-flask level resulted in the production of 1.5–1.6 g/L natamycin [15, 16]. Li et al. [20] were able to produce about 10.38 g/L natamycin in shake-flask cultivations with the addition of lower alcohols. On the other hand, combining butanol addition with random mutation resulted in the production of 1.62 and 2.8 g/L natamycin in shake-flask and bioreactor levels, respectively [18]. We have previously investigated the effect of addition of short-chain carboxylic acids on the production of natamycin in both shake-flask and bioreactor levels. The optimized cultivation and addition conditions resulted in the production of 3.0 and 3.98 g/L natamycin in both cultivation systems, respectively [5].

The production of polyene macrolides is limited by the application of glucose as a carbon source due to its integration in the antibiotic molecule [25]. However, glucose is a fast assimilated C-source and is known to repress the production of many antibiotics due to carbon catabolite regulation [26]. Despite prolonged industrial interest in natamycin, relatively little information has been published regarding production processes under the influence of glucose limitation.

In the present work, the effect of initial glucose concentration on the kinetics of cell growth, glucose consumption and natamycin production was investigated. Moreover, the effect of intermittent feeding of glucose or glucose-beef extract mixture on natamycin production was investigated in shake-flask cultivations. Finally, the process of natamycin production was studied in 7.5 L-stirred tank bioreactor under batch and continuous feeding conditions.

## Results

### Effect of initial glucose concentration on kinetics of cell growth and natamycin production in batch cultures

**Fig. 1:**
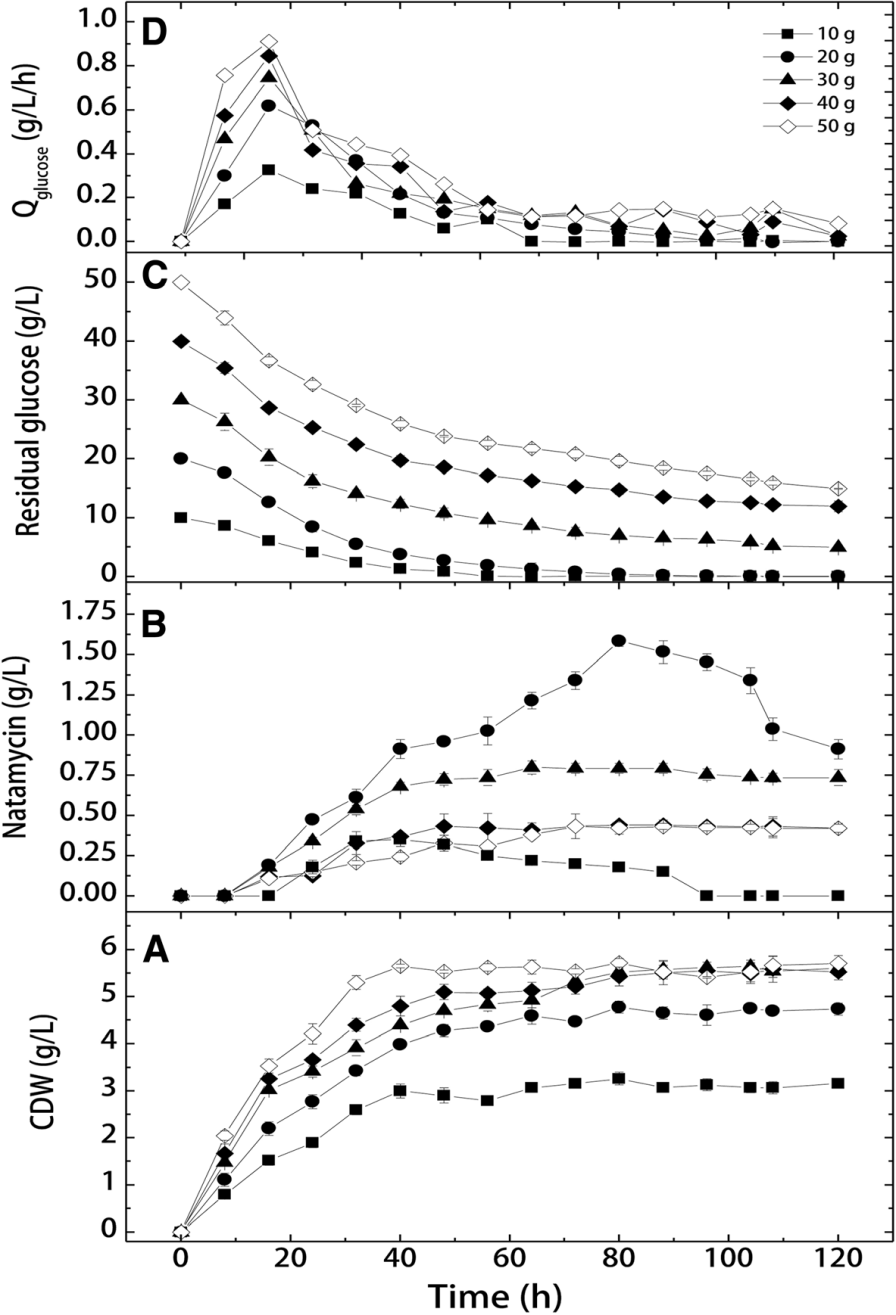
Effect of different initial glucose concentrations on cell growth and natamycin production *by S. natalensis* (CDW, cell dry weight; Q_glucose_, glucose consumption rate)

These experiments aimed at investigating the effect of different initial glucose concentration on cell growth and production kinetics in shake flask cultivations. The obtained results (Fig. 1, Table 1) clearly showed that initial glucose concentration significantly affected production process parameters (*p* = 0.001). Under all glucose concentrations, cells grew exponentially for the first 40 h, where they reached their maximal cell dry weight. After which, cells entered the stationary phase for the rest of the cultivation time. It can be seen that both maximal cell dry weight and cell growth rates increased proportionally with the initial glucose concentration. At 50 g/L glucose, a maximal cell dry weight of 5.66 ± 0.19 g/L was obtained, which was 73.6% higher than X_max_ obtained at 10 g/L. Furthermore, it can be seen that average cell growth rates at 20, 30, 40 and 50 g/L increased by about 33.0, 46.7, 60.0 and 88.0%, respectively, than the Q_x_ obtained at 10 g/L. Additionally, the maximal cell dry weights obtained at 30–50 g/L recorded comparable values by the end of cultivation (Table 1).

**Table 1:** Kinetic parameters of cell growth and natamycin production by *S. natalensis* NRRL 2651 as affected by initial glucose concentration

Concentration (g/L)	X_max_ (g/L)	P_max_ (g/L)	ΔS (%)	Q_x_^a^ (g/L/h)	Q_p_^a^ (g/L/h)	Q_s_^a^ (g/L/h)	*Y*_x/s_ (g/g)	*Y*_p/s_ (g/g)	*Y*_p/x_ (g/g)
10	3.26 ± 0.13	0.35 ± 0.04	100	0.075	0.011	- 0.22	0.35	0.038	0.121
20	4.78 ± 0.13	1.58 ± 0.03	100	0.099	0.021	- 0.41	0.28	0.083	0.332
30	5.66 ± 0.14	0.82 ± 0.03	85.8	0.11	0.013	- 0.39	0.39	0.044	0.185
40	5.59 ± 0.23	0.43 ± 0.02	72	0.12	0.006	- 0.38	0.31	0.027	0.122
50	5.66 ± 0.19	0.43 ± 0.07	70	0.14	0.006	- 0.50	0.31	0.015	0.102

X_max_: maximal cell dry weight; P_max_: maximal natamycin production; ΔS: % consumed glucose; Q_X_: average cell growth rate; Q_P_: average natamycin production rate; Q_S_: average glucose consumption rate; *Y*_X/S_: maximal yield of biomass per consumed glucose; *Y*_P/S_: maximal yield of natamycin per consumed glucose; *Y*_P/X_: maximal yield of natamycin per biomass. ^a^ Data are calculated at the end of the exponential growth phase

For glucose consumption, results showed that both 10 and 20 g/L glucose cultivations were characterized by complete glucose consumption at an average consumption rate of − 0.22 and − 0.41 g/L/h after 40 and 80 h, respectively. On the other hand, cultivations supplemented with higher glucose concentration (Q_s_) did not suffer from glucose depletion, and their average glucose consumption rates increased with increasing glucose concentrations (− 0.44, − 0.51 and − 0.60 g/L/h, for 30, 40 and 50 g/L, respectively). By the end of the cultivation time, about 16.7, 29.8 and 29.9% of the initial glucose remained in the cultivation media supplemented with 30, 40 and 50 g/L glucose, respectively.

Concerning natamycin production, results showed that natamycin production started at all glucose concentrations after 8 h. At 10 g/L glucose, natamycin was produced with an average production rate of 0.011 g/L/h and reached its maximal volumetric production of 0.35 ± 0.04 g/L after 40 h. Increasing glucose concentration to 20 g/L has a pronounced effect on natamycin production, where its production rate increased by about 91% (0.02 g/L/h) and reached a maximal production of 1.58 ± 0.032 g/L after 80 h. On the other hand, further increase in glucose concentration (≥ 30 g/L) adversely affected natamycin production where the maximal volumetric productivity decreased by about 48.1, 72.8 and 72.8% at 30, 40 and 50 g/L, respectively, from the maximal production at 20 g/L. Moreover, the average production rate at these cultivations decreased and ranged from 0.006 to 0.013 g/L/h. The collective data in Table 1 shows that the growth and production coefficient yields recorded their maximal values at 20 g/L glucose. Furthermore, at 20 g/L, it can be seen that the substrate conversion yield into biomass was the least recorded (0.28 g cells/g consumed glucose), while natamycin yields per consumed glucose and produced cell mass were at their maximal values (0.083 g natamycin/g consumed glucose, and 0.33 g natamycin/g cells).

**Fig. 2:**
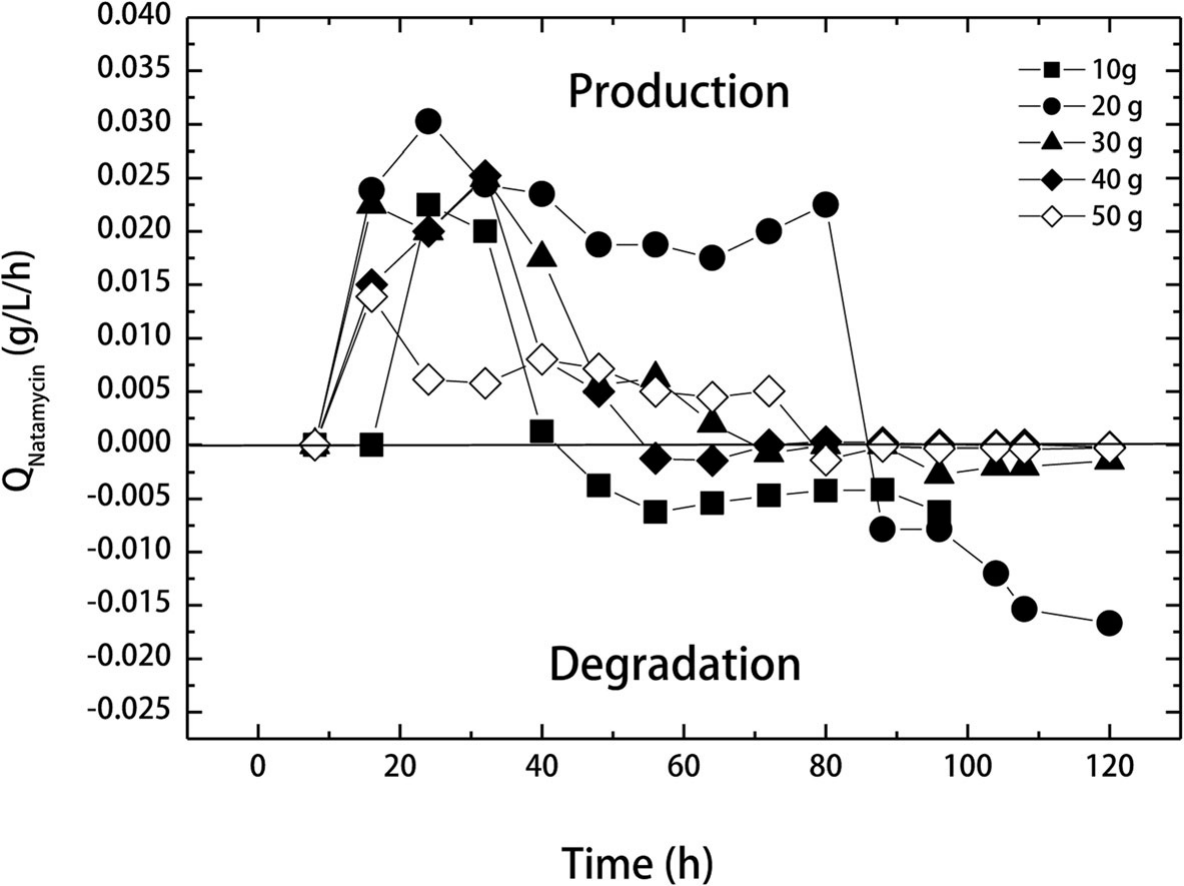
Production and degradation rates of natamycin in batch culture as affected by different initial glucose concentrations (Q_natamyccin_, natamycin production rate)

It can be further observed that at 10 and 20 g/L glucose the natamycin production decreased gradually with time reaching a minimum of 0.15 ± 0.02 and 0.913 ± 0.06 g/L at 88 and 120 h, respectively. Furthermore, in cultivation with 20 g/L glucose, natamycin completely disappeared in culture broth after 96 h, in other words, 16 h after complete glucose consumption. Therefore, the data representing the progress of natamycin production rates at different initial glucose concentrations were summarized in Fig. 2. It can be seen that the production rates increased initially at all glucose concentrations, and then started to slow down. At 10 and 20 g/L glucose, natamycin production rates started to decrease after 40 and 80 h and recorded negative rate values (− 0.0053 and − 0.0143 g/L/h, respectively) indicating that the produced natamycin was degraded in the medium (rates below the base line). On the other hand, at higher glucose concentrations, natamycin production rates decreased and were almost around the zero level (− 0.0012, − 0.0004 and − 0.0007 g/L/h at 30, 40 and 50 g/L, respectively), meaning that natamycin production was stopped. In such case, the produced natamycin still can be determined in the production medium at all three concentrations (30–50 g/L).

### Effect of intermittent feeding of glucose-beef extract mixture on cell growth and natamycin production by *S. natalensis* in shake flasks

**Fig. 3:**
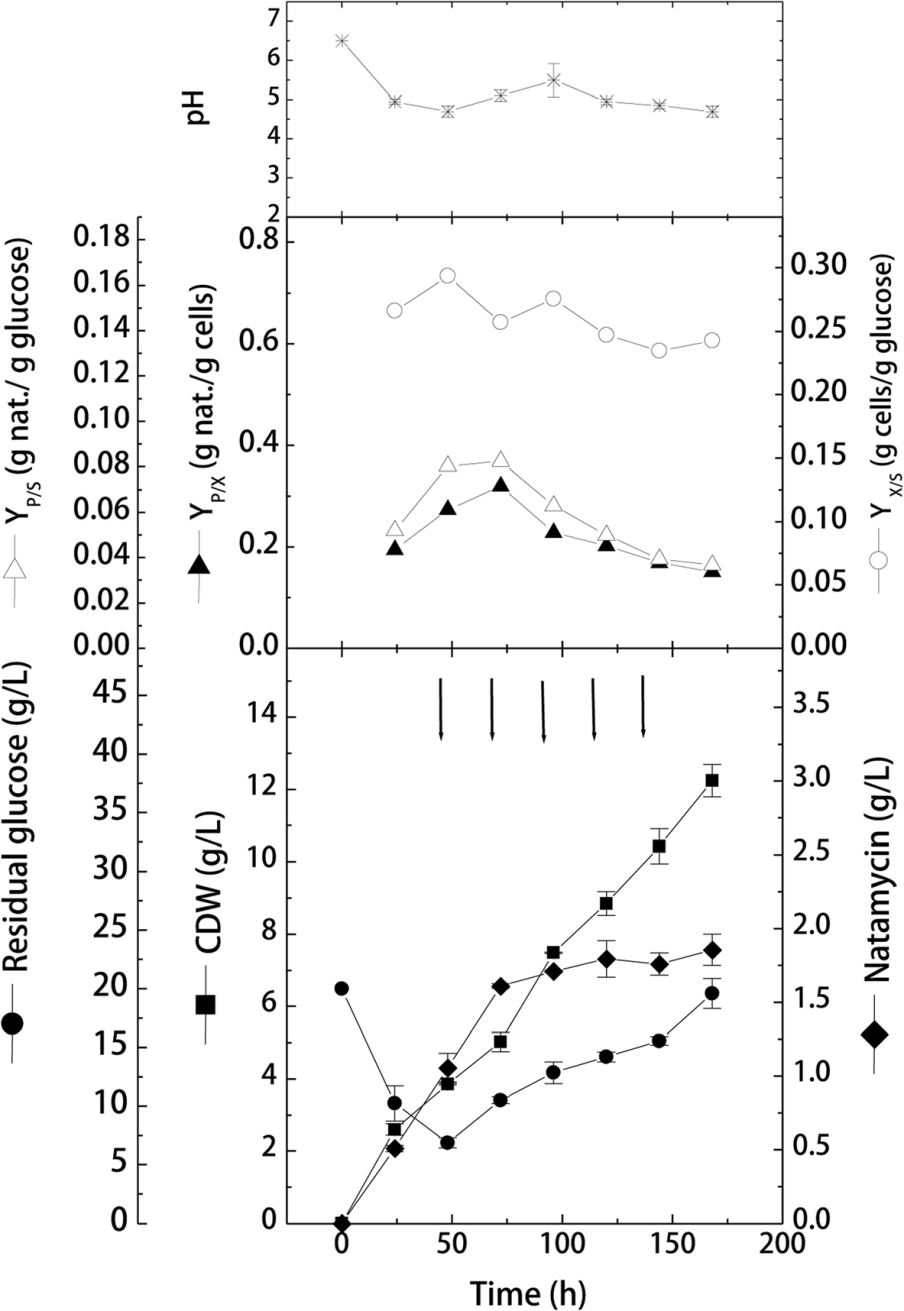
Effect of intermittent feeding of glucose and beef extract on cell growth and natamycin production by *S. natalensis* in shake-flask cultivation (CDW, cell dry weight; *Y*_X/S_: yield of biomass per consumed glucose; *Y*_P/S_: yield of natamycin per consumed glucose; *Y*_P/X_: yield of natamycin per biomass)

During these cultivations, a sterile feeding solution containing glucose and beef extract was fed to the growing cells at the same initial C:N ratio (before glucose depletion). Feeding started after 48 h of cultivation and was repeated every 24 h for 5 repeated batches. Obtained results (Fig. 3) showed that cells grew exponentially for the first 48 h with an average growth rate of 0.08 g/L/h, reaching 3.86 ± 0.021 g/L, and consumed glucose at an average consumption rate of − 0.27 g/L/h. Upon intermittent addition, cells continued to grow almost with the same growth rate (0.07 g/L/h) and reached a maximal cell growth of 12.25 ± 0.45 g/L at 168 h. However, during intermittent feeding of glucose-beef extract mixture, the average glucose consumption rate was increased by about 1.15-folds (− 0.311 g/L/h). Consequently, glucose started to accumulate in the production medium with each feeding batch, where a final residual concentration of 19.6 ± 1.27 g/L of glucose was found by the end of the cultivation (39% of the total added glucose).

For natamycin production, it can be seen that during the first normal batch (48 h), natamycin was produced by a normal batch production rate of 0.022 g/L/h, where volumetric production reached 1.057 ± 0.09 g/L. However, after the first feeding batch (at 72 h), natamycin concentration recorded 1.61 ± 0.02 g/L, a 20% increment from the 1.34 ± 0.05 g/L obtained in normal batch cultivation after 72 h (Fig. 1). After the 2nd feeding batch (at 96 h), natamycin concentration reached 1.709 ± 0.009 g/L, which corresponds to 17.5% increase from its corresponding batch value (1.454 ± 0.05 g/L at 96). Although batch data (Fig. 1) showed that natamycin started to decompose in the production medium after 88 h, and reached a final of 0.913 ± 0.06 g/L by 120 h, however, the intermittent feeding significantly improved natamycin production (*p* = 0.02), where a final concentration of 1.855 ± 0.11 g/L was obtained at 168 h.

Yield coefficient results showed that *S. natalensis* cells continued to produce natamycin after the 1st addition batch with yield coefficients similar to those reported in the batch cultivation (*Y*_*x/s*_, 0.26 g cells/g consumed glucose; *Y*_*P/s*_, 0.083 g natamycin/g consumed glucose; *Y*_*P/X*_, 0.32 g natamycin/g cells). However, yield coefficients decreased with further addition batches.

### Effect of intermittent feeding of glucose on cell growth and natamycin production by *S. natalensis* in shake flasks

**Fig. 4:**
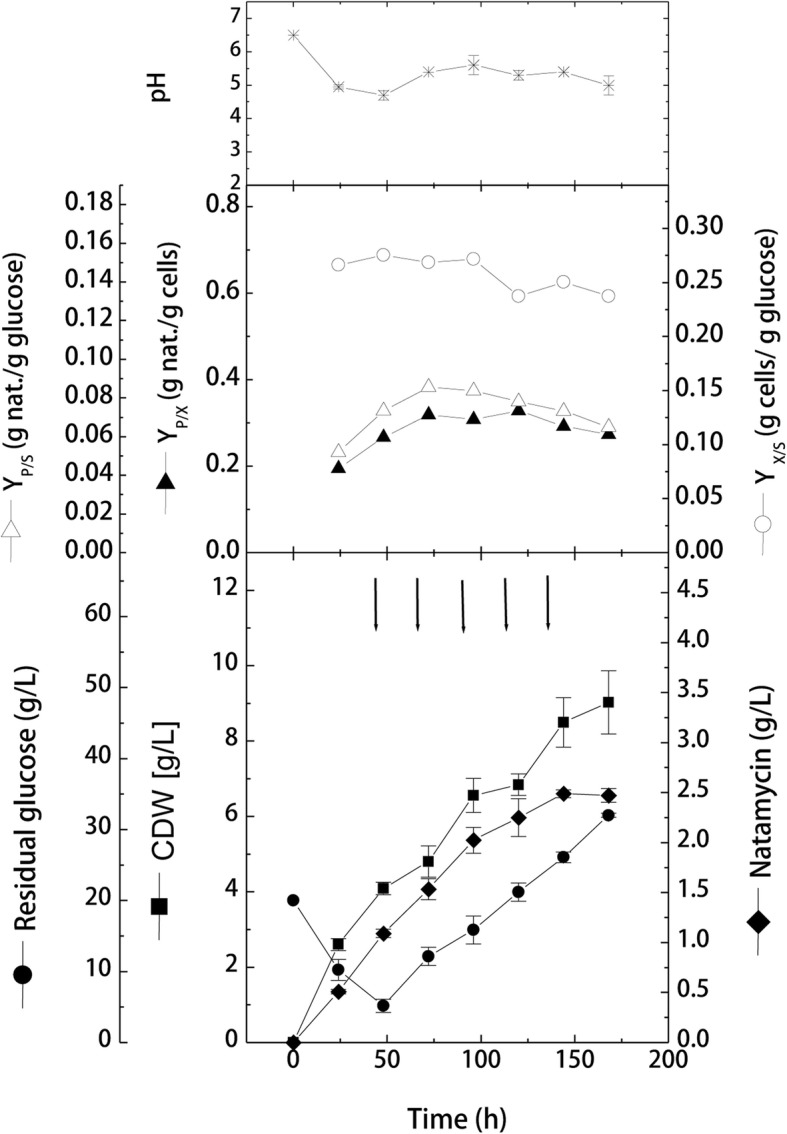
Effect of intermittent feeding of glucose on cell growth and natamycin production by *S. natalensis* in shake-flask cultivation (CDW, cell dry weight; *Y*_X/S_: yield of biomass per consumed glucose; *Y*_P/S_: yield of natamycin per consumed glucose; *Y*_P/X_: yield of natamycin per biomass)

This part of the work was designed to investigate the effect of intermittent feeding of glucose only on the kinetics of cell growth and natamycin production. The cultivation was performed as previously described, except that the feeding solution contained only glucose at a concentration of 10 g/L. Results obtained (Fig. 4) showed that, similar to previous batch experiment, cells grew with an average growth rate of 0.085 g/L/h and a glucose consumption rate of − 0.31 g/L/h reaching 4.09 ± 0.16 g/L at 48 h. After starting the intermittent glucose feeding phase, cells continued to grow exponentially for the rest of the cultivation. However, it can be seen that the average growth rate decreased to 0.04 g/L/h, which is about 42.9% lower than the average growth rate recorded in case of feeding glucose-beef mixture (0.07 g/L/h). This lower growth rate was reflected on the maximal cell growth obtained by the end of cultivation (9.03 ± 0.82 g/L), which was lower by about 26.3% from the maximal growth obtained in glucose-beef feeding.

During batch -wise glucose feeding phase, the average glucose consumption decreased from − 0.31 to − 0.19 g/L/h, which is about 60% of the average glucose consumption rate obtained during batch-wise glucose-beef feeding (− 0.31 g/L/h). This decrease in substrate consumption rate highly increased glucose accumulated in the cultivation flasks, where it recorded 32 ± 0.29 g/L at 168 h, representing about 64% of the total fed glucose.

Looking at natamycin production kinetics, it can be observed that after the 1st addition batch, natamycin production increased by 1.4-folds recording 1.533 ± 0.11 g/L, and then increased with each glucose addition until reaching a maximal of 2.49 ± 0.04 g/L at 144 h. Additionally, it can be seen that after the 1st glucose addition, natamycin production increased by about 18, 25.7, 41.5 and 33.3% after the 2nd, 3rd, 4th and 5th addition, respectively, than those concentrations obtained after the corresponding glucose-beef additions. It is further worth to mention that the maximal produced natamycin in this experiment (2.49 ± 0.04 g/L) was 1.57-folds higher that the maximal concentration obtained in normal shake flask batch (1.58 ± 0.03 g/L). Yield coefficient results showed that *Y*_*P/s*_ was slightly improved from 0.083 to 0.086 g natamycin/g consumed glucose for glucose-beef and glucose addition, respectively, suggesting that more glucose was converted into natamycin.

### Cell growth and natamycin production 7.5 L-stirred tank bioreactor under batch cultivation mode

**Fig. 5:**
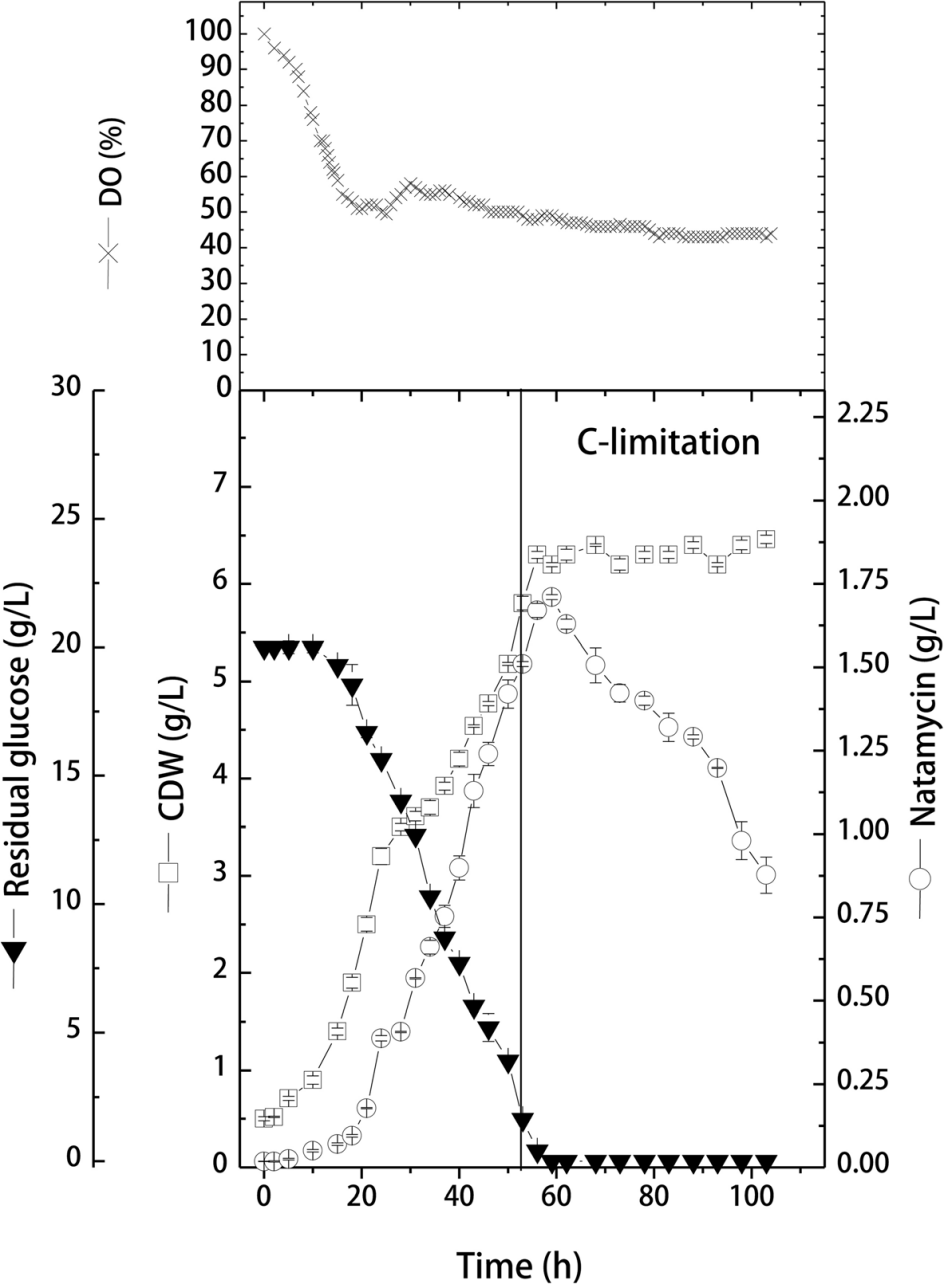
Cell growth and natamycin production by *S. natalensis* in 7.5 L-stirred tank bioreactor under batch cultivation mode (CDW, cell dry weight; DO, dissolved oxygen)

This part of the work aimed at investigating the effect of transferring natamycin production process from shake-flask level into stirred tank bioreactor level. *S. natalensis* cells were inoculated into 7.5 L-stirred tank bioreactor with 5 L working volume. The cultivation was firstly performed under batch conditions to monitor cell behavior and production kinetics. Results obtained (Fig. 5) showed that bioreactor cultivation significantly improved kinetics of cell growth and natamycin production in comparison to shake-flask batch cultivation (*p* = 0.003). During the exponential growth phase, cells grew with an average growth rate of 0.111 g/L/h, and reached 6.3 ± 0.04 g/L of cell growth. Although cells grew almost with the same growth rate recorded in shake-flask cultivation (0.099 g/L/h), however, in bioreactor cultivation, the exponential growth phase was extended to 56 h (14 h longer than that in shake-flask) before entering the stationary phase. This longer active growth was reflected on the higher cell concentration obtained before the onset of the stationary phase (1.97-folds higher than cell concentration in shake-flask culture). The dissolved oxygen (DO) concentration started to decrease sharply with time up to 20 h, at an average DO consumption rate of − 2.5%/h. After 20 h, DO concentration remained more or less constant until the end of cultivation, meaning that dissolved oxygen was not limiting. A final cell growth of 6.46 ± 0.04 g/L was obtained at 103 h.

Furthermore, in bioreactor cultivation, the average glucose consumption rate increase by about 23% (− 0.443 g/L/h) than that recorded in shake-flask cultivation (− 0.36 g/L/h). Such higher consumption rate can also be seen from the highest yield coefficient (*Y*_*x/s*_), which recorded 0.73 g cells/g consumed glucose (0.28 g cells/g consumed glucose in shake-flask cultivation).

Natamycin was produced during the stationary growth phase with an average production rate of 0.038 g/L/h, 1.8-folds higher than that recorded in shake-flask cultivation. Consequently, a maximal natamycin production of 1.71 ± 0.01 g/L was obtained after 59 h. However, similar to shake-flask cultivation, it can be seen that natamycin degradation started directly after glucose depletion (60 h), while in shake-flask cultivation, degradation started after 80 h. Also, in both cultivations, the natamycin degradation rates were similar (− 0.014 and − 0.019 g/L/h for shake-flask and bioreactor cultivations, respectively).

### Kinetics of cell growth, glucose consumption and natamycin production by *S. natalensis* during fed-batch cultivation in 7.5 L-stirred tank bioreactor

**Fig. 6:**
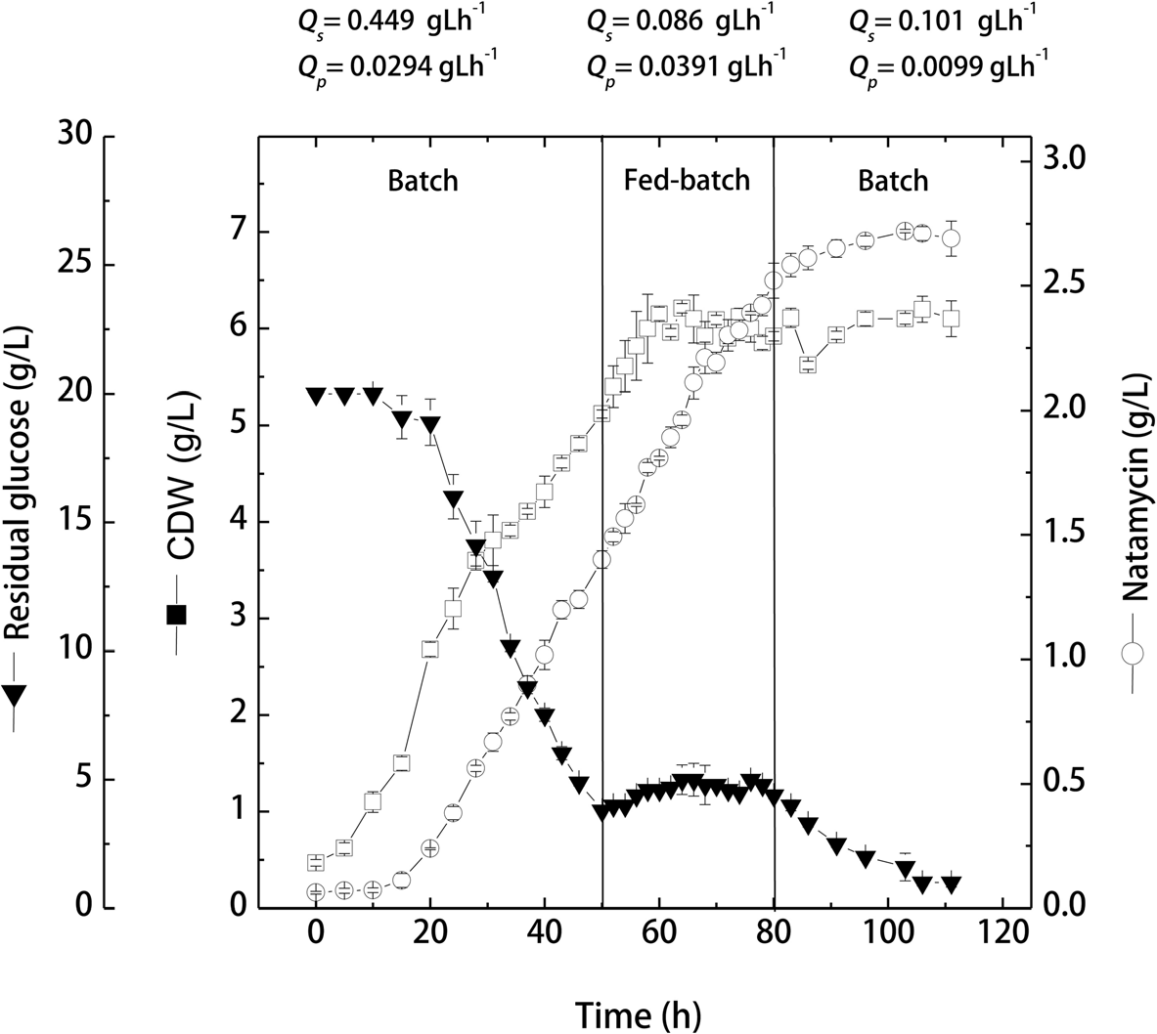
Kinetics of cell growth, glucose consumption and natamycin production by *S. natalensis* during fed-batch cultivation in 7.5 L-stirred tank bioreactor (CDW, cell dry weight)

The fed-batch bioreactor cultivation was designed to investigate the effect of continuous glucose feeding on the kinetics of cell growth and natamycin production in order to overcome the problems of glucose limitation. After inoculation, the cultivation was run as normal batch for the first 50 h. Based on the glucose consumption rate obtained from the bioreactor batch run (− 0.443 g/L/h), sterile glucose solution was continuously fed to the bioreactor at a feeding rate of 0.5 g/L/h. Feeding was started shortly before glucose becomes limited (at 3.8 g/L), and this phase lasted for the next 30 h. After 80 h, feeding was stopped and the cultivation mode was turned again into batch mode. Results (Fig. 6) showed that the first batch session proceeded as normal batch where cells reached 5.12 ± 0.04 g/L with a natamycin production of 1.4 ± 0.035 g/L. During this phase, growth and production kinetic parameters were similar to a great extent to those obtained in the previous batch cultivation.

Upon feeding glucose to the production medium, cells continued to grow exponentially for the first 14 h of feeding with a similar growth rate (0.103 g/L/h), and reached their maximal growth of 6.21 ± 0.05 g/L at 64 h. Afterwards, cell growth entered the stationary phase, and remained more or less constant. During the feeding phase, glucose consumption remained constant, where no accumulation was observed and the average available glucose concentration ranged from 4.0 ± 0.07 to 4.4 ± 0.04 g/L. Therefore, natamycin production continued during the feeding phase, where the average production rate increased up to 0.04 g/L/h (34.5 and 95.7% higher than the production rate in bioreactor and shake-flask batch cultivations, respectively). Consequently, natamycin production reached 2.52 ± 0.07 g/L by the end of the feeding phase, and continued to be produced during the second batch phase (at a smaller production rate), where a maximal production of 2.72 ± 0.01 g/L was obtained at 103 h. It can be seen that feeding increased maximal natamycin by 1.6- and 1.72-folds from the maximal production obtained in bioreactor and shake-flask batch cultivations, respectively.

The production yield coefficients in terms of consumed glucose and producing cells (*Y*_*p/s*_, *Y*_*p/x*_) were significantly improved in response to continuous glucose feeding. The *Y*_*p/x*_ increased from 0.28 g natamycin/g cells (at 50 h) reaching a maximal of 0.42 g/g by 80 h, and increased further during the 2nd batch phase to reach 0.47 g/g at 86 h. Similarly, *Y*_*p/s*_ increased from 0.09 g natamycin/g consumed glucose reaching its maximal at 80 h (0.16 g/g, 2-folds the yield before feeding).

Finally, Table 2 summarized different kinetic parameters obtained for cell growth and natamycin production under different cultivation conditions. It can be seen that the final cultivation in bioreactor under glucose feeding conditions significantly (*p* = 0.003) improved process parameters in terms of cell growth, substrate consumption, and natamycin production kinetics.

**Table 2:** Summarized kinetic parameters of cell growth and natamycin production by *S. natalensis* NRRL 2651 under different cultivation conditions applied

Parameter	Cultivation conditions				
	Shake-flask cultivation			Bioreactor cultivation	
	Batch (20 g/L)	Intermittent feeding (glucose+beef)	Intermittent feeding (glucose)	Batch (20 g/L)	Glucose feeding
X_max_ (g/L)	4.780 ± 0.13	12.25 ± 0.45	9.030 ± 0.84	6.460 ± 0.04	6.21 ± 0.05
P_max_ (g/L)	1.580 ± 0.03	1.855 ± 0.1	2.480 ± 0.04	1.710 ± 0.01	2.72 ± 0.01
Q_x_^a^ (g/L/h)	0.099	0.080	0.085	0.111	0.103^b^
Q_p_^a^ (g/L/h)	0.021	0.022	0.023	0.038	0.045^b^
Q_s_^a^ (g/L/h)	−0.41	−0.31	−0.220	−0.443	−0.448
*Y*_x/s_ (g/g)	0.280	0.260	0.280	0.760	0.61
*Y*_p/s_ (g/g)	0.083	0.083	0.086	0.090	0.162
*Y*_p/x_ (g/g)	0.332	0.320	0.330	0.280	0.46

Xmax: maximal cell dry weight; Pmax: maximal natamycin production; QX: average cell growth rate; QP: average natamycin production rate; QS: average glucose consumption rate; YX/S: maximal yield of biomass per consumed glucose; YP/S: maximal yield of natamycin per consumed glucose; YP/X: maximal yield of natamycin per biomass. a Data are calculated at the end of the exponential growth phase. b Data are taken during the feeding phase

## Discussion

Obtained results showed that glucose concentration has a pronounced effect on natamycin production by *S. natalensis*. Increasing glucose concentration up to 20 g/L increased natamycin production, and further increase in its concentration had remarkably decreased the concentration of natamycin. Furthermore, cell growth kinetics, glucose consumption and natamycin production rates, as well as yield coefficients, showed their maximal values at 20 g/L. These results are in accordance with those previously reported [5, 15], where highest natamycin concentration were obtained at lower glucose concentrations. Additionally, higher glucose concentrations showed higher cell growth levels. This can be explained on the basis of carbon catabolite repression and feedback regulation of higher glucose concentrations. Generally, actinomycetes respond negatively towards higher concentrations of glucose and similar low molecular readily metabolizable carbon sources [25]. Higher glucose concentrations usually stimulate higher growth and retarded the production of secondary metabolites. It has been reported that readily utilizable sugars repress or inhibit the enzymatic machinery responsible for the biosynthesis of many antibiotics, e.g. puromycin, kanamycin, penicillin and cephalosporin [27, 28].

Recently, it has been postulated that carbon catabolite repression regulates glucose metabolism by the phosphoenolpyruvate-dependent phospotransferase system affecting glucose kinase (Glk), which acts only on glucose and converts it into glucose-6-phosphate [25, 29]. It has been found that unknown glucose metabolites activate Glk, and that there is an unknown regulatory factor that binds to Glk and changes its function from catabolic one into regulatory one, leading finally to carbon catabolite repression phenomenon [30].

Furthermore, our results showed that natamycin concentrations decreased in 10–20 g/L glucose cultivations, while cultivations at higher glucose concentrations did not show such decrease in the volumetric natamycin production. This can be attributed to the cleavage of natamycin molecule by growing cells, present under glucose limitation or depletion, resulting in the release of glucose moiety from the macrolactone ring, and making it available for cells as maintenance energy [5, 15]. Similar results were reported on the decrease in natamycin concentration after reaching its maximal production [2, 18]. However, in both cases, the decrease in production was not attributed to natamycin hydrolysis, since the cultivation media contained other slowly metabolized carbon sources. Our results are also in good agreement with those reported by Marin and McDaniel [31], who suggested the same explanation for the decrease obtained in their production of candicidin after 70 h, when glucose was depleted from the medium. Additionally, it has been reported that amphotericin B has been used as an endogenous substrate by the producing cells [32]. In such case, the produced antibiotic was transformed into the inactive form. The same authors found non-active forms of polifungin and candihexin lacking mycosamine moiety in the fermentation broth following glucose depletion, suggesting the degradation or modification of such antibiotics by the producing cells.

Another possible explanation for the decrease in natamycin concentration at higher glucose concentrations (30–50 g/L), is the fast glucose metabolism and the production of organic acids, which in turn decrease the pH of the medium. It is well known that pH shift towards acidic values results in the cleavage of aminosugar moiety of mycosamine and its release from the natamycin molecule [33]. Moreover, the propensity to autolysis is a well-known property of different actinomycetes in prolonged cultivations. This has been attributed to C-source depletion, which activates certain molecular mechanism, i.e. proteolytic cascades and nucleases, which in turn results in decreased cell proliferation, differentiation and cell death [34–36].

To overcome the carbon catabolite repression in actinomycetes, it is recommended to perform glucose cultivations with slow feeding of carbon source. Therefore, the second part of the work was conducted to investigate the effect of glucose feeding on the kinetics of cell growth and natamycin production. Firstly, intermittent repeated feeding of glucose alone or glucose-beef extract mixture was carried out on shake-flask levels. Results showed that both intermittent feeding strategies improved the production process in comparison to the preliminary batch cultivation. Volumetric natamycin production increased by about 1.3- and 1.57-folds in glucose-beef mixture or glucose feeding, respectively, than the corresponding batch cultivation. This suggests that slow feeding is useful to bypass the carbon catabolite repression regulation in *Streptomyces* in case of using glucose as a sole carbon source. Slow feeding of glucose allows higher respiration rates during antibiotic production phase (idiophase) as well as higher productivity [32]. Sugar addition has also been used for the increased production of other polyene antibiotics to overcome rapid glucose consumption [37, 38].

However, results showed that glucose feeding was superior to the feeding of glucose-beef mixture in terms of natamycin production. Also, cell growth was much higher in case of glucose-beef feeding. This can be accredited to the fact that the addition of beef extract tends to increase the concentration of organic nitrogen in the production medium. This will result in the conversion of the production phase (idiophase) into growth phase (trophophase), where the metabolism is directed towards cell growth and consequently lowered antibiotic production [28, 31]. This was accompanied by higher glucose consumption in the glucose-beef feeding, and therefore, lower amounts of glucose were accumulated by the end of the cultivation. Periodic addition of nitrogen source with the fed glucose increased growth over candidin production [28, 39]. Furthermore, the increase in cell growth in case of nitrogen source feeding can be due to the increase in phosphate content found in beef extract. Generally, complex nitrogen sources are used for their balanced contents of fatty acids, proteins, cations and low phosphate. However, the accumulation of beef extract in the medium can lead to increased levels of phosphate in the production medium, which in turns inhibits natamycin production. Phosphate effect on the production of antibiotics by streptomycetes is well known and reported [25, 40].

The production process was transferred to 7.5 L-stirred tank bioreactor to investigate the possibility of scaling-up the process from shake-flask into semi-industrial bioreactor level. Results showed that maximal natamycin production was reached at the onset of the stationary phase accompanied by complete glucose exhaustion. Additionally, natamycin production increased linearly and proportional to cell growth. Bioreactor cultivation is generally characterized by the presence of more controlled conditions in terms of pH, better oxygenation and mixing due to controlled aeration and agitation. Such conditions provide the growing cells with more adequate conditions promoting better growing environments and allowing maximal metabolic cellular activities [41, 42]. Our results also showed that oxygen consumption increased sharply for the first 20 h, representing cell growth in trophophase, and then oxygen consumption remained more or less stable for the rest of cultivation. These results are in agreement with those of Wang et al. [17], who reported on the enhancement of natamycin production by genetically manipulated *S. gilvosporeus*. They reported similar oxygen consumption rates for the first 12–20 h of cultivation, where the progress of cell growth was at its highest rates.

The batch bioreactor results showed also decreased concentrations of natamycin after glucose depletion from the cultivation medium. Accordingly, continuous feeding of glucose was carried out in bioreactor to investigate the kinetics of cell growth and natamycin production under these optimized conditions. Results showed that continuous glucose feeding significantly improved cell growth and natamycin production. Volumetric production increased by 1.6- and 1.72-folds from the concentration obtained in batch cultivations in bioreactor and shake-flasks, respectively. This was accompanied by improved specific production yields based on glucose consumption and growing cells. Also, glucose catabolite repression and product degradation were avoided and cells continue to grow and produce natamycin until the end of the cultivation. Furthermore, substrate consumption and natamycin production rates were kept nearly constant during batch and fed-batch phases. Although feeding glucose has not yet been explored for natamycin production, however, previous reports are found for the increased production of other polyene antibiotic using slow glucose feeding [28, 31]. Authors suggested that direct or indirect glucose metabolites are involved in the switch between primary and secondary metabolism. Generally, fed-batch cultivation is the choice for improving antibiotic production processes [21–23]. It is used in the production of secondary metabolites, which strictly require fast metabolizable carbon sources in order to avoid their fast metabolism and the conversion from idiophase into torphophase [37, 38]. Furthermore, Wang et al. [43] reported on the production of isovaleryispiramycin under glucose limitation by investigating the effect of different glucose concentrations on the activity of the rate limiting enzyme in the biosynthetic pathway, branched-chain α-keto acid dehydrogenase (BCKDH). They found that glucose depletion in the late production phase significantly decreased the activity of the BCKDH and consequently isovaleryispiramycin concentration. They suggested glucose feeding during this phase to keep glucose levels between 0.0 and 1.0 g/L, which significantly improved enzyme activity and hence, isovaleryispiramycin production.

## Conclusions

From the aforementioned results, it can be inferred that initial glucose concentration significantly affects natamycin production through activation or inhibition of carbon catabolite repression mechanism. Slow glucose feeding was found to support better natamycin production and prevents the degradation of the product due to glucose limitation. Furthermore, the addition of glucose and beef extract shifted the glucose metabolism and the cultivation from the idiophase into the trophophase, favoring cell growth and retarding natamycin production. Finally, 7.5 L-stirred tank bioreactor was used to optimize the fed-batch cultivation for natamycin production. Such fed-batch resulted in the production of 2.72 ± 0.01 g/L of natamycin after 103 h, which corresponds to an increase of about 1.6- and 1.72-folds from the maximal production obtained in bioreactor and shake-flask batch cultivations, respectively.

## Methods

### Microorganism and inoculum preparation

*Streptomyces natalensis* (NRRL 2651) obtained from the National Center for Agricultural Utilization Research (NRRL, Peoria, Illinios, USA), was used throughout this work. Inoculum in the form of spores obtained from a densely sporulated culture grown on ISP agar medium for 7 days at 30 °C was used [5]. The arisen spores were harvested with a sterile physiological saline solution (0.9% NaCl, w/v), and were counted directly using a haemocytometer slide. The natamycin production medium was inoculated with spore suspension at a final concentration of 1 × 10^7^ spores/mL.

### Cultivation media

ISP agar medium [16], used for sporulation, was composed of (g/L) as follows: Malt extract (Difco), 10.0; Glucose (BDH), 4.0; and Yeast extract (Difco), 4.0. The final pH was adjusted to 7.2 before sterilization. Unless otherwise stated, natamycin production medium was composed as follows (g/L): Glucose (Difco), 20.0; Yeast extract (Difco), 2.0; Beef extract (Difco), 8.0 and DL-Asparagine (Fluka), 0.5. The final pH was adjusted to 7.2 before sterilization. Glucose was sterilized separately and added to the medium directly before inoculation. Cultivations were carried out using Erlenmeyer flasks with a total volume of 250 mL containing 50 mL medium. The flasks were cultivated on a rotary shaking incubator at 30 °C and 200 rpm (New Brunswick Scientific Co., Edison, New Jersey, USA).

### Bioreactor cultivation

Submerged cultivations were carried out in a 7.5 L stirred tank bioreactor Microferm (MF-107, New Brunswick Scientific Co., New Brunswick, NJ, USA) with a working volume of 5 L. Sterilization was carried out at 121 °C for 30 min. and the separately sterilized glucose was added after cooling. The bioreactor vessel was inoculated as previously described [15], and agitation was performed using a three 4-bladed rushton turbine impellers (*d*_*i(impeller diameter)*_ = 48 mm; *d*_*t(tank diameter)*_ = 143 mm, *d*_*i*_*d*_*t*_^*− 1*^ = 0.34) at 600 rpm. Aeration was performed by filtered sterile air (1 v/v/m). Dissolved oxygen concentrations were analyzed by polarographic electrode (Ingold, Germany). The pH was adjusted to 7.2 by the addition of 2.5 M NaOH and controlled during the cultivation. Foam was suppressed, when necessary, by the addition of silicon antifoam reagent (Fluka, Switzerland).

### Feeding cultivations on shake-flask and bioreactor levels

This section was designed to evaluate the effect of feeding glucose (or glucose + beef extract) on the kinetics of cell growth and natamycin production by *S. natalensis* NRRL 2651. In shake-flask cultures, after inoculation and cell growth for 48 h, 5 mL of sterilized feeding solution (10 g/L glucose, or 10 g/L glucose + 4 g/L beef extract) were added to the growing cells under aseptic conditions. The feeding solution of glucose and beef extract was prepared at the same C:N ratio in the initial cultivation medium. Intermittent feeding was repeated every 24 h for 5 batches. At the end of each addition run, representative samples (2 flasks) were withdrawn and necessary analyses were performed. In case of bioreactor cultivations, cells were allowed to grow in a batch mode for 50 h, and then sterilized glucose solution was continuously fed at a feeding rate of 0.5 g/L/h (depending on the rate of glucose consumption calculated form the bioreactor batch experiments). Continuous glucose feeding was conducted for 30 h, and then the bioreactor cultivation mode was switched again to normal batch mode. Samples were taken and assayed at regular intervals.

### Analyses

#### Sample preparation

During cultivation progress, samples were periodically withdrawn (a: shake flasks: 3 flasks, each 50 mL broth, or b: bioreactor: 10 mL broth). The volume of the samples was determined using pre-weighed sterile Falcon centrifuge tubes (BD Biosciences, Franklin Lakes, NJ, USA), and samples were then centrifuged at 5000 rpm for 20 min. A small fraction of the supernatant was frozen at − 20 °C for glucose and natamycin determination.

#### Determination of cell dry weight

The remaining pelleted cells in the Falcon tubes were washed thoroughly using saline solution, and were then centrifuged again. The final supernatant was discarded and the cell pellet was allowed to dry overnight at 80 °C until a constant weight was reached [44]. The final obtained cell dry weight was expressed as g/L.

#### Determination of natamycin concentration

The determination of natamycin concentration in the fermentation broth was carried out by two methods. Firstly, the fermentation broth was assayed biologically by standard agar plate diffusion method using *Saccharomyces cerevisiae* as the test organism [5]. Aliquots of the cultivation broth were loaded into wells bored in agar plates, which were previously seeded with the test organism at standard assay conditions. Plates were incubated at 30 °C. The diameters of the inhibition zones were recorded after 24 h. A biological standard curve was drawn between the logarithms of different concentrations of standard natamycin (Gist-Brocades, Delft, Netherlands) and the inhibition zone.

For confirmation, natamycin concentrations were determined using HPLC (Agilent 1200, Agilent Technologies, USA) as described in Wang et al. [17]. The HPLC system was equipped with a UV detector (303 nm) and a Zorbax Eclipse C18 column. The mobile phase contained NH_4_Cl: acetonitrile: tetrahydrofuran (75:20:5) and it was run at a flow rate of 0.5 mL/min. A standard natamycin (Gist-Brocades, Delft, Netherlands) calibration curve was used to determine natamycin concentration.

#### Determination of glucose concentration

The concentration of residual glucose in the medium was detected spectrophotometrically. A glucose enzyme determination kit (Biocon Diagnostik GmbH, Burbach, Germany) was used.

#### Calculation of kinetic parametrs

1 $$ {\mathrm{Q}}_{\mathrm{x}}\;\mathrm{Cell}\kern0.17em \mathrm{growth}\kern0.17em \mathrm{rate}\;\left(\mathrm{g}/\mathrm{L}/\mathrm{h}\right)=\frac{X_2-{X}_1}{t_2-{t}_1} $$

where *X*_2_ and *X*_1_ are cell mass concentrations at time *t*_2_ and *t*_1_, respectively.

2 $$ {\mathrm{Q}}_{\mathrm{s}},\mathrm{Glucose}\kern0.17em \mathrm{consumption}\ \mathrm{rate}\;\left(\mathrm{g}/\mathrm{L}/\mathrm{h}\right)=\frac{C_2-{C}_1}{t_2-{t}_1} $$

where *C*_2_ and *C*_1_ are glucose concentrations at time *t*_2_ and *t*_1_, respectively.

3 $$ {\mathrm{Q}}_{\mathrm{P}},\mathrm{Natamycin}\kern0.17em \mathrm{production}/\mathrm{degradation}\kern0.17em \mathrm{rate}\;\left(\mathrm{g}/\mathrm{L}/\mathrm{h}\right)=\frac{P_2-{P}_1}{t_2-{t}_1} $$

where *P*_2_ and *P*_1_ are natamycin concentrations at time *t*_2_ and *t*_1_, respectively.

#### Statistical analysis and result reproducibility

Experiments were repeated thrice to ensure result reproducibility. In case of shake flask cultures, three flasks were simultaneously withdrawn at the same interval, and their contents were assayed separately. Results were analyzed using SPSS 9.0, and were expressed as mean ± SD for three replicates. ANOVA one-way analyses were used to analyze results, and statistical significance was defined as *p* < 0.05.

## Data Availability

All the data generated in this current work are included in the ‘Result and Discussion’.

## Abbreviations

ANOVA: Analysis of variance
